# Safety assessment of antibiotic and probiotic feed additives for *Gallus gallus domesticus*

**DOI:** 10.1038/s41598-017-12866-7

**Published:** 2017-10-16

**Authors:** D. P. Neveling, L. van Emmenes, J. J. Ahire, E. Pieterse, C. Smith, L. M. T. Dicks

**Affiliations:** 1 Department of Microbiology, University of Stellenbosch, Private BagX1, Matieland, 7602 Stellenbosch South Africa; 2 Department of Animal Science, University of Stellenbosch, Private BagX1, Matieland, 7602 Stellenbosch South Africa; 3 Department of Physiological Sciences, University of Stellenbosch, Private BagX1, Matieland, 7602 Stellenbosch South Africa

## Abstract

Antibiotics in feed select for resistant strains and is thus a threat to human health. In this study, the effect of a multi-strain probiotic and antibiotics on the growth and health of broilers was studied. Equal numbers of broilers received on a daily basis either a multi-strain probiotic or a combination of sulphadiazine, colistin and trimethoprim, whereas the control group received standard feed. The villi of immature broilers (19 days old) administered antibiotics had a larger surface area and their lymphocyte and basophil counts were higher compared to broilers from the probiotic and control groups. The cecal microbiomes of mature broilers (29 days old) that received probiotics had higher levels of *Enterobacteriaceae*, but lower numbers of Clostridiales, *Brucellaceae*, *Synergistaceae*, *Erysipelotrichaceae* and *Coriobacteriaceae* compared to the antibiotic-treated group. A decline in the bioluminescence of *Listeria monocytogenes* observed for broilers on probiotics suggested that the probiotic may be used to control bacterial infections. No significant differences in total red blood cell, haemoglobin and haematocrit content, and mean values for corpuscular volume, corpuscular haemoglobin and corpuscular haemoglobin numbers were recorded amongst broilers from the different treatment groups. This study provides valuable information on the health and performance of broilers when administered probiotics and antibiotics as additives.

## Introduction

Poultry reared on a large scale in intensive production systems are more prone to develop microbial infections^1^. Necrotic enteritis, caused by *Clostridium perfringens* and coccidiosis, caused by *Eimeria* spp., are the most challenging of all poultry diseases and are difficult to control^2^. The use of antibiotics as growth promoter in animal feed has been banned by the European Union in an attempt to control natural selection for antibiotic-resistant pathogens^3^ and ensure that currently available antibiotics remain effective in the treatment of animal and human infections.

The general assumption is that chickens intensively reared do not acquire beneficial microbiota from the environment^4^. Furthermore, the immune system of broilers, especially in the first month, is not well developed and they are susceptible to bacterial infections caused by *Campylobacter jejuni*, *C. perfringens*, *Salmonella enterica* and *Escherichia coli* ^5^. It is thus not surprising that broilers reared intensively are more susceptible to microbial infections^6^. Those that do survive the first two weeks have a good chance to develop a stable consortium of intestinal microbiota during the following two weeks^7^.

Alternative supplements that enhances growth and protect broilers from pathogenic infections is desperately needed. Numerous beneficial effects of probiotics administered to broilers have been reported, e.g. improvement in growth performance^8,9^, increased digestion of nutrients^10^, modulation of intestinal microflora^11^, inhibition of pathogens^12,13^, competitive exclusion of pathogens and antagonism^14^, and modulation of gut mucosal immunity^15^. However, the addition of probiotics to broiler feed is still far from being implemented on a regular basis^16^, mainly due to a lack in in-depth knowledge about the complex dynamics of the poultry gut^17^ and the multitude of parameters that influences the efficacy of probiotics. Differences in microbial species and strains, methods used to propagate probiotic strains, differences in the ability of the strains to adhere to the gastro-intestinal tract (GIT), number of evidence-based clinical trials^18^, production standards^19^, environmental factors and management^6^ are a few of the variabilities. More research on the intestinal ecosystem, and inter-microbial and microbiota-host interactions are required^16^.

In this study we evaluated the effect of antibiotics (sulphadiazine, colistin and trimethoprim in combination) and a multi-strain probiotic (*L*. *crispatus*, *L*. *salivarius*, *L*. *gallinarum*, *L*. *johnsonii*, *E*. *faecalis* and *B*. *amyloliquefaciens*) on the performance of healthy broilers. Parameters assessed included growth performance, immune organ weight, gizzard weight, histomorphology of the small intestine, haematology, tibia bone mineral weight, inhibition of *L. monocytogenes* EGDe *in vivo* and changes in cell numbers of cecal microorganisms. Understanding what physiological changes these feed additives induce in healthy broilers is important to assess their safety with long term use.

## Results and Discussion

### Health and growth performance

The average feed conversion ratios (FCRs) of broilers in the probiotic and antibiotic treatment groups were not significantly different from that recorded for broilers in the untreated group on days 7, 14, 21 and 28 (Supplementary Table S1), suggesting that neither the multi-strain probiotic, nor the antibiotics had an effect on growth performance. Similar results were published by Gheisar and co-workers^20^. The authors reported an increase in body weight of broilers that received *Enterococcus faecium* M74, but their FCRs were not significantly different from broilers in control groups on a probiotic-free diet^20^. However, studies conducted by Shim *et al*.^21^, Sinol *et al*.^22^ and Chen *et al*.^23^, using different probiotic compositions, showed an improvement in growth performance. The authors attributed enhanced growth to an increase in digestive enzyme activity, coupled to additional changes, such as a decrease in ammonia production and maintenance of beneficial microbiota in the GIT. In a recent study where broilers were fed a multi-strain probiotic consisting of *L*. *johnsonii, L*. *crispatus*, *L*. *salivarius* and an unidentified *Lactobacillus* sp., no changes in body weight gain (BWG), feed intake (FI) and FCR were observed^24^. Similar conclusions were drawn when a multi-strain probiotic, consisting of hetero- and homofermentative *Lactobacillus* spp., were administered to broilers^25^.

The inconsistency in reports regarding the effect of probiotics on growth performance may be due to differences in rearing conditions, strain compositions, number of viable cells administered and frequency of administration. Santos and co-workers^26^ have shown differences in growth performance when broilers were reared free-ranged, on an open floor, and in cages. Cage rearing is considered more hygienic, as broilers are not in direct contact with faeces^27^. However, cage rearing could also lead to food-safety concerns due to the inadequate transfer of beneficial microbiota from the environment^26^. Rearing conditions should thus always be taken into account when the effect of additives on growth performance is studied^28^. In the present study, broilers were reared in cages elevated from the floor.

Probiotic characteristics are strain dependent and the combination of strains may have an effect on the efficacy of a multi-strain probiotic^29^. The number of viable cells administered and the dose frequency are equally important. Most probiotics are administered at 10^7^ to 10^9^ cfu per day^30^. In the present study, broilers received between 1.0 × 10^8^ and 4.1 × 10^8^ cfu of the multi-strain probiotic per day. Antibiotics and probiotics act as prophylactics that inhibit the development of pathogenic bacteria and improves growth. However, our results indicated that the daily administration of a multi-strain probiotic (10^8^ cfu) did not have a positive, nor negative, effect on growth performance.

### Haematology, organ weight and histology

Several factors, such as physiological and environmental conditions^31^, diet^32^, water and feed restriction^33^, age^34^ and administration of drugs^35^ affect the blood parameters of healthy broilers. At day 19 the white blood cell (WBC), heterophil (HET), monocyte (MONO) and eosinophil (EOS) counts were not significantly different for broilers from the different treatment groups (Table 1). Lymphocyte (LYM) and basophil (BASO) counts, on the other hand, were significantly different at day 19. Broilers from the antibiotic treatment group had a higher mean LYM (p = 0.012) and BASO (0.018) count compared to the probiotic treatment group. LYM and BASO counts were not significantly different between probiotic and control, and antibiotic and control treatment groups at day 19 (Table 1). Lymphocytes include natural killer cells, T-cells and B-cells^36^. T cells (thymus cells) and B-cells (bone marrow- or bursa-derived cells) are the major cellular components of the adaptive immune response. T-cells are involved in cell-mediated immunity, whereas B-cells are primarily responsible for humoral immunity^36^. Natural killer cells are part of the innate immune system and play a major role in defending the host from tumours and virus-infected cells^36^. Basophils are granulocytes responsible for inflammatory responses and production of heparin and histamine^36^. A higher BASO count is characteristic of a pro-inflammatory response and may be the result of sensitivity to antibiotics or the presence of bacteria that elicits an immune response. Transiently higher LYM counts at day 19 were indicative of a response to the presence of specific immune provoking bacteria. These counts usually normalises when bacterial cell numbers are brought under control, or after a few days when the body develops tolerance to the antibiotics. Both these responses are undesired. As energy for growth is relayed to elicit an immune response, probiotics showed the opposite and did not elicited an immune response, which is desirable. At day 29, no significant differences were recorded in WBC, HET, LYM, MONO, EOS and BASO counts for broilers from the different treatment groups (Table 1).

**Table 1:** Mean (±standard deviation) total leukocyte count (WBC) at day 19 and 29, and composition of heterophils (HET), lymphocytes (LYM), monocytes (MONO), eosinophils (EOS) and basophils (BASO) of broilers receiving different treatments.

Treatment	WBC (10^6^/µl)		HET (10^3^/µl)		LYM (10^3^/µl)		MONO (10^3^/µl)		EOS (10^3^/µl)		BASO (10^3^/µl)	
	19 D	29 D	19 D	29 D	19 D	29 D	19 D	29 D	19 D	29 D	19 D	29 D
**Control**	34.5 ± 26.5	27.9 ± 21.3	5.8 ± 2.5	7.2 ± 2.4	23.9^ab^ ± 20.8	18.1 ± 18.5	1.9 ± 1.9	0.75 ± 0.74	0.12 ± 0.04	0.17 ± 0.12	1.44^ab^ ± 1.53	0.49 ± 0.48
**Antibiotic**	49.5 ± 33.5	29.5 ± 27.3	6.3 ± 2.5	5.9 ± 3.5	38.7^a^ ± 27.8	17.6 ± 18.9	3.1 ± 2.6	0.55 ± 0.60	0.18 ± 0.11	0.16 ± 0.10	2.74^a^ ± 1.65	0.64 ± 0.97
**Probiotic**	21.2 ± 11.9	32.6 ± 21.9	4.7 ± 2.6	7.3 ± 2.8	13.9^b^ ± 8.0	23.3 ± 20.3	1.4 ± 1.3	0.68 ± 0.65	0.10 ± 0.06	0.16 ± 0.12	1.09^b^ ± 1.04	0.64 ± 0.67
**p value**	0.07	0.832	0.414	0.281	**0.040** ^*^	0.621	0.168	0.698	0.708	0.950	**0.046** ^*^	0.820

^*^p < 0.05, ^a, b^Means within columns with different superscripts differ significantly (p < 0.05).

Lymphocytes are the major circulating immune cells in birds and HET are functionally equivalent to neutrophils that participate in inflammation and phagocytosis. A high HET/LYM ratio and high glucocorticoid level is an indication of stress^37,38^. High HET/LYM ratios have also been associated with increased mortality^39^. No significant differences were recorded between the mean HET/LYM ratios of broilers from the different treatment groups at day 19 (p = 0.737) and day 29 (p = 0.357) (Supplementary Table S2). Thrombocytes stop bleeding by clumping and plugging injured blood vessels. No significant differences were recorded in thrombocyte counts for the different treatment groups at day 19 (p = 0.121) and day 29 (p = 0.350) (Supplementary Table S2).

At day 19, broilers from the different treatment groups had no significant differences in total red blood cell (RBC), haemoglobin content (HGB), haematocrit content (HCT), mean corpuscular volume (MCV), mean corpuscular haemoglobin (MCH) and mean corpuscular haemoglobin counts (MCHC); listed in Table 2. The erythrocyte cell distribution (RDW) of broilers from different treatment groups at day 19 were significantly different (p = 0.022). Broilers receiving the multi-strain probiotic had a higher mean RDW at day 19, compared to the antibiotic (p = 0.033) and control (p = 0.009) groups. No significant differences were recorded between the antibiotic and control treatment groups. Higher RDW levels may be due to older age of RBC, mixed deficiency (iron, B12 or folate), recent haemorrhage and false positive results from EDTA anticoagulated blood^40,41^. Results suggest that probiotic-treated broilers may in some way allow for RBC to age further before being recycled, as RDW increases with cell age. The relatively small RDW changes supports this interpretation, as the differences would have been larger in the event of deficiency or haemorrhage. Broilers receiving different treatments at day 29 had no significant differences in their RBC, HCT, MCV, MCH, and MCHC and RDW counts.

**Table 2:** Mean (±standard deviation) erythrocyte count and haemoglobin content (HGB), haematocrit value (HCT), mean corpuscular volume (MCV), mean corpuscular haemoglobin (MCH), mean corpuscular haemoglobin concentration (MCHC) and erythrocytes cell distribution width (RDW) of broilers receiving different treatments.

Treatment	**RBC (10** ^**6**^ **/µl)**		**HGB (g/dl)**		**HCT (%)**		**MCV (f)**		**MCH (pg)**		**MCHC (g/dl)**		**RDW (%)**	
	19 D	29 D	19 D	29 D	19 D	29 D	19 D	29 D	19 D	29 D	19 D	29 D	19 D	29 D
**Control**	2.22 ± 0.26	2.59 ± 0.34	13.24 ± 1.71	14.75 ± 1.83	18.33 ± 2.12	20.49 ± 2.85	82.56 ± 2.85	81.17 ± 2.84	59.62 ± 1.86	58.31 ± 2.17	72.24 ± 2.02	71.88 ± 2.84	12.70^bc^ ± 0.85	12.56 ± 0.63
**Antibiotic**	2.19 ± 0.28	2.27 ± 0.74	13.22 ± 1.83	12.56 ± 5.11	18.39 ± 2.16	19.10 ± 4.73	84.06 ± 2.71	81.33 ± 3.47	60.24 ± 1.33	58.08 ± 1.33	71.72 ± 2.57	71.44 ± 2.70	12.93^b^ ± 1.08	12.67 ± 1.26
**Probiotic**	2.17 ± 0.45	2.46 ± 0.30	10.88 ± 3.21	14.24 ± 2.12	17.76 ± 3.78	19.41 ± 2.33	81.64 ± 2.40	79.26 ± 2.39	52.43 ± 20.55	57.04 ± 2.42	64.57 ± 25.86	72.01 ± 3.48	13.76^a^ ± 0.41	12.81 ± 0.97
**p value**	0.960	0.174	0.056	0.135	0.861	0.549	0.140	0.106	0.301	0.223	0.475	0.888	**0.022** ^*^	0.794

^*^p < 0.05, ^a, b c^Means within columns with different superscripts differ significantly (p < 0.05).

Bursa of Fabricius and the spleen are lymphoid organs which forms part of the avian immune system^42^. The spleen filters and regenerates antibodies, whereas the bursa of Fabricius is the site of haematopoiesis responsible for B-cell production. Immune organ weights are weighed to evaluate the immune status of broilers^23^. The bursa of Fabricius is the primary lymphoid and probiotic administration can lead to an increase in weight^43^, which may be considered an improvement of the immune system^44^, however, excessive responses depress growth performance^45^. Administration of either the multi-strain probiotic or antibiotics did not alter the relative weights of the spleen, bursa of Fabricius and the spleen: bursa of Fabricius ratio at days 19 and 29 (Supplementary Table S3). Conflicting results were reported for the probiotic Protexin® (*Lactobacillus plantarum*, *Lactobacillus bulgaricus*, *Lactobacillus acidophilus*, *Lactobacillus rhamnosus*, *Bifidobacterium bifidum*, *Streptococcus thermophilus*, *Enterococcus faecium*, *Aspergillus oryzae* and *Candida pintolopesii*)^28,46^. Pourakbari *et al*.^28^ observed no differences in spleen or bursa weights of broilers raised in cages, but Dizaji *et al*.^46^ reported an increase in spleen weights when broilers were raised on the floor. Concluded from these studies, differences in rearing conditions, i.e. housing, feed composition and environmental factors, probably played an important role for the observed discrepancies. In our study the multi-strain probiotic (*L*. *crispatus* DPN167, *L*. *salivarius* DPN181, *L*. *gallinarum* DPN164, *L*. *johnsonii* DPN184, *E*. *faecalis* DPN94 and *B*. *amyloliquefaciens* DPN123) was administered to broilers reared in cages, which could be the reason why no differences in immune organ weights were observed. It may thus be more applicable to study the effect of the multi-strain probiotics on broilers raised under less hygienic conditions, as cage rearing is considered more hygienic than pen rearing^27^.

Relative gizzard to body weight ratio is used to assess the efficiency of mechanical feed digestion. Supplementation of feed with either the multi-strain probiotic or antibiotics had no significant effect on the relative gizzard weights at days 19 and 29 (Supplementary Table S4). Researchers using a multi-strain probiotic which consisted of *L*. *acidophilus*, *Lactobacillus casei*, *Pediococcus acidilactici*, *Bacillus subtilis* and *Saccharomyces boulardii* ^47^ and a single strain probiotic *Eubacterium* sp.^48^ similarly reported no significant differences in the relative gizzard weights.

The surface of the small intestine contains villi that increases the surface area and leads to increased absorption. At the base of the villi, tubular invaginations (crypts) extend into connective tissue to form enterocytes (absorptive cells). A decrease in villi height leads to a reduction in surface area and reduces the absorption of nutrients^49^. The ratio between villi height and crypt depth is used as an indicator of digestive capacity. A low ratio correlates to decreased digestion and absorption^3^. Deeper crypt depths correlates with increased absorption^50^. However, shorter villi and deeper crypts may decrease absorption and increase endogenous losses through loss of enterocytes, thus leading to a decrease in absorption^51^. The villus height, crypt depth and villus to crypt depth ratios of broilers from the different treatment groups were not significantly different at day 19 (Table 3). However, the mean villi area at day 19 for the different treatments were significantly different (p = 0.042). Broilers from the untreated group had larger villi area compared to the antibiotic (p = 0.029) and probiotic (p = 0.026) treatment groups. No significant differences were recorded between the probiotic and antibiotic treatment groups. Larger villi areas leads to larger surface areas and increased absorption of nutrients^52^. However, increased villi area could also be considered detrimental for nutrient absorption. If villi height are the same for two treatment groups (as in the current study), but the area per villus is larger in the one treatment group, the number of villi per cm^2^ are less and so also the total surface area for nutrient absorption. Numerous anatomical characteristics affect the absorptive capacity, i.e. tract length, villus height and width, and the number of villi per unit area all contribute to absorptive capacity^53^. The chicks in our study were day old as-hatched and from the same genetic lineage, representing a homogenous collection of broilers with similar anatomical characteristics. Taken together, this suggests that both the antibiotic and multi-strain probiotic treatment groups had better absorption capacity when compared to the control group at this time point. As observed for haematological parameters, this difference was also transient, as the villus height and area, crypt depth and villus to crypt depth ratios were similar for the three groups at day 29 (Table 3). The effects of probiotics on villus surface area seems to depend on the segment in which the bacteria colonizes^52^. For example, researchers assessing *B. subtilis* LS 1–2^54^ and GalliPro® - which consist of *B*. *subtilis* DSM 17299^55^ - found an increase in the villus height, surface area and villus height-to-crypt depth ratio. This highlights the requirement for probiotic-specific assessments, as well as comprehensive analyses of various segments of the GIT before firm conclusions can be made.

**Table 3:** Mean (±standard deviation) villi height, villi area, crypt depth and villus height: crypt depth ratio of the duodenum of broilers slaughtered on day 29.

**Treatment**	Villi Height (µm)		Villi Area (µm)		Crypt depth (µm)		Villi height: crypt depth	
	19 D	29 D	19 D	29 D	19 D	29 D	19 D	29 D
Control	275 ± 21	1522 ± 90	8332^a^ ± 1448	215439 ± 55696	45 ± 10	240 ± 23	6.30 ± 1.20	6.40 ± 0.69
Antibiotic	275 ± 39	1496 ± 153	6810^b^ ± 1333	214644 ± 49877	42 ± 10	232 ± 27	6.87 ± 1.78	6.53 ± 0.95
Probiotic	260 ± 20	1515 ± 170	6573^b^ ± 1073	216848 ± 48577	40 ± 8	219 ± 21	7.32 ± 1.75	6.99 ± 0.98
**p value**	0.556	0.949	**0.042** ^*^	0.952	0.298	0.213	0.659	0.449

^*^p < 0.05, ^a, b^Means within columns with different superscripts differ significantly (p < 0.05).

### Mineralization of the tibia

The degree of bone mineralisation affects bone strength, phosphorus and/or calcium deficiencies and lead to an increase in bone breakage and defects^56^. This influences animal welfare, growth performance and meat quality^57^. Tibia bone weight and ash weight is used to evaluate bone mineralisation^58^. Probiotics support calcium absorption primarily by the production of metabolites, enzymes and vitamins, some of which participate in the metabolism of calcium^59^. Broilers from the different treatment groups showed no significant differences in their tibia bone weights, or bone ash percentages at day 29 (Supplementary Table S5) and administration of either the multi-strain probiotic or antibiotic combination did not alter bone mineralization efficiency.

### Inhibition of *L*. *monocytogenesin vivo*

**Figure 1:**
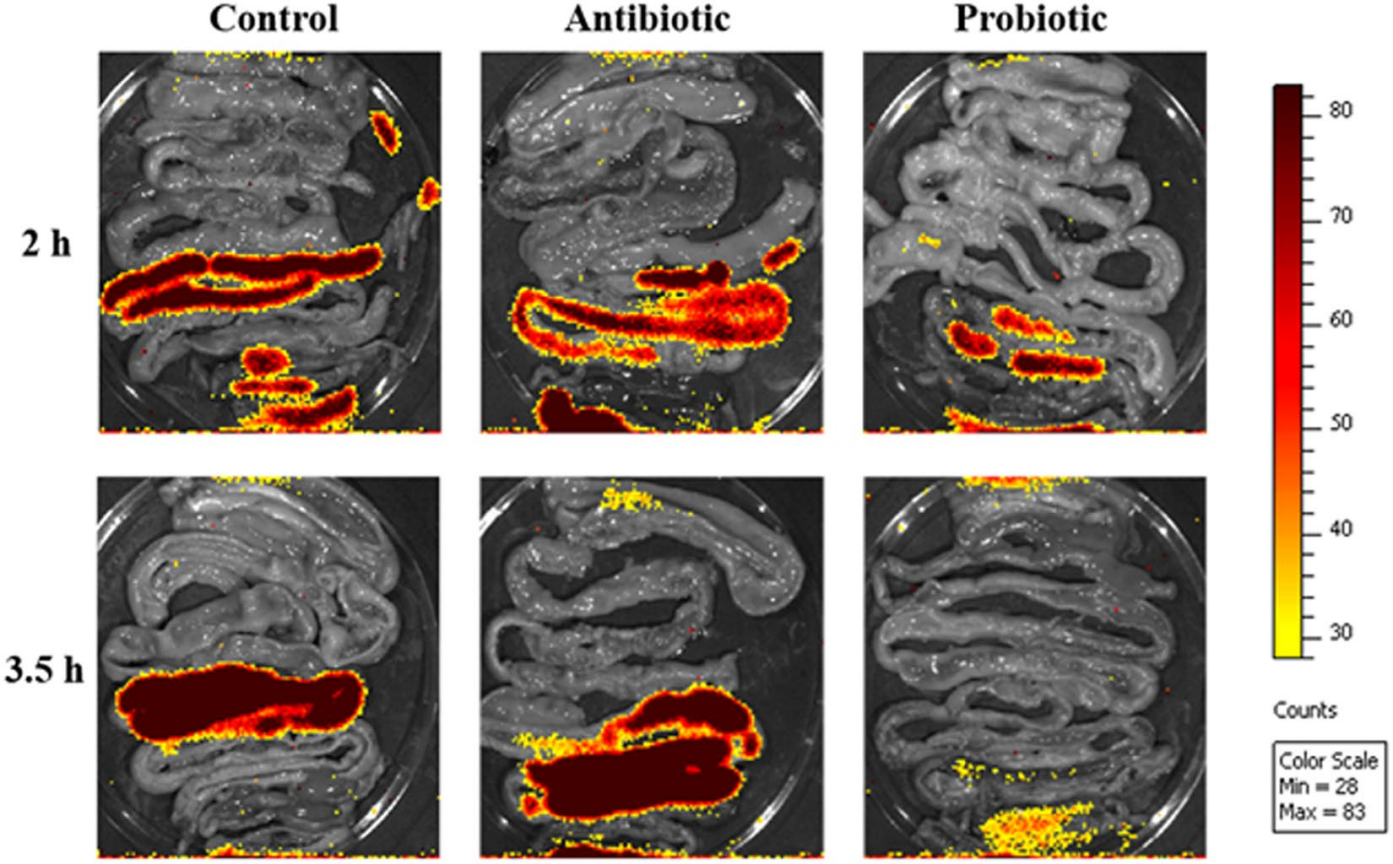
Bioluminescent images of isolated gastro-intestinal tracts of broilers from the different treatments groups (i.e. multi-strain probiotic, antibiotic combination and control) at 2 and 3.5 h after administration of bioluminescent *L*. *monocytogenes* EGDe (4.2 × 10^8^ cfu).

**Figure 2:**
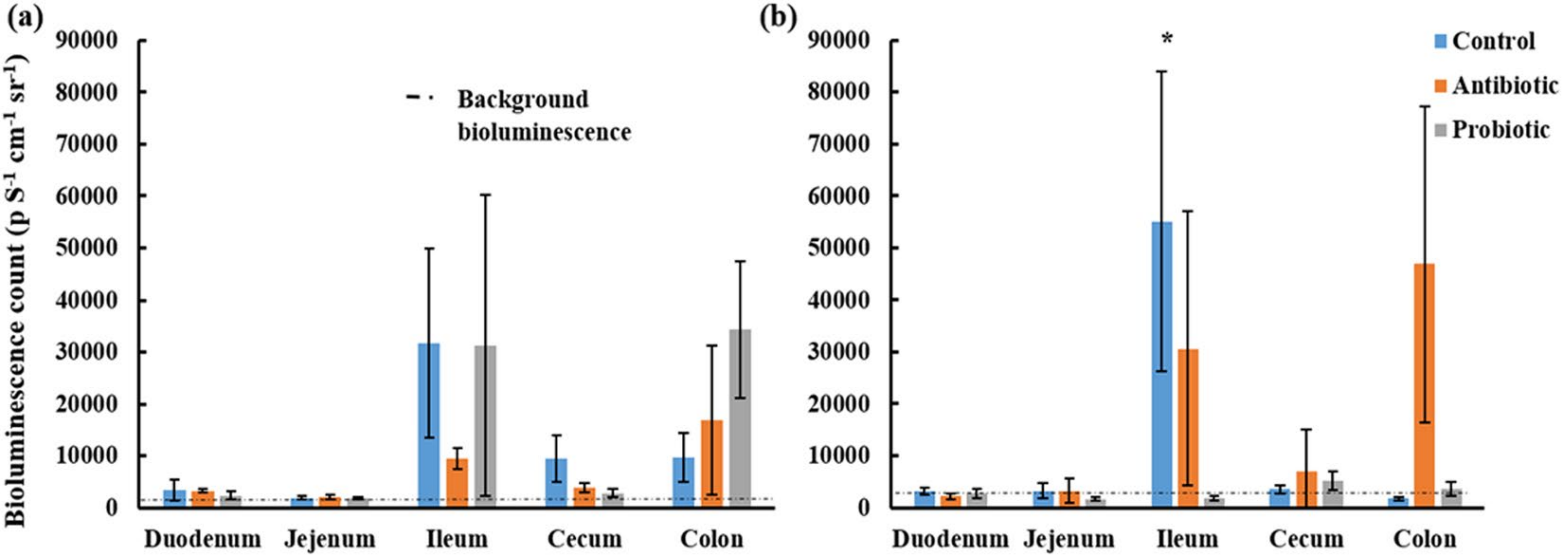
Bioluminescence counts (p S^−1^ cm^−1^ sr^−1^) for the different gastro-intestinal compartments (duodenum, jejunum, ileum, cecum and colon) of broilers from the different treatment groups (multi-strain probiotic, antibiotic combination and control) at 2 and 3.5 h after administration of *L*. *monocytogenes* EGDe. *Indicates significant differences (p < 0.05; Kruskal-Wallis nonparametric test).

Bioluminescent *L*. *monocytogenes* was administered to broilers to determine whether the antibiotic and probiotic feed additives could inhibit colonization and proliferation of the pathogenic bacterium *in vivo*. Transition of bioluminescent *L*. *monocytogenes* EGDe in the gastrointestinal tract of broilers from the different treatment groups, after 2.0 and 3.5 h, is shown in Fig. 1. Lower levels of bioluminescence were observed in the GIT of broilers from the probiotic treatment group after 3.5 h, compared to broilers from the control and antibiotic groups. High levels of bioluminescence were observed in the ileum and colon, and low levels in the duodenum and cecum (Fig. 1). Broilers from the control group showed high bioluminescent readings (mean p.S^−1^.cm^−1^.sr^−1^) in the ileum (3.17 × 10^4^) and low readings in the duodenum (3.46 × 10^3^), cecum (9.48 × 10^3^) and colon (9.71 × 10^3^) after 2 h (Fig. 2). After 3.5 h, high readings were observed in the ileum (5.5 × 10^4^) and low readings in the duodenum (3.19 × 10^3^), jejunum (3.2 × 10^3^) and cecum (3.52 × 10^3^). Broilers from the antibiotic treatment group had high bioluminescence readings in the colon (1.69 × 10^4^), and low levels in the duodenum (3.28 × 10^3^), jejunum (2.07 × 10^3^), ileum (9.45 × 10^3^) and cecum (3.82 × 10^3^) after 2 h (Fig. 2). After 3.5 h, high bioluminescence readings were observed in the ileum (1.01 × 10^5^) and colon (4.69 × 10^4^), and low readings in the duodenum (2.21 × 10^3^), jejunum (3.21 × 10^3^) and cecum (6.93 × 10^3^). For probiotic-treated broilers, high readings were observed in the ileum (3.13 × 10^4^) and colon (3.43 × 10^4^), and low readings in the duodenum (2.32 × 10^3^) and cecum (2.84 × 10^3^) at 2 h. After 3.5 h, low levels were observed in the duodenum (2.79 × 10^3^), cecum (5.14 × 10^3^) and colon (3.62 × 10^3^). Decrease in bioluminescence observed in the probiotic treatment group after 3.5 h suggests that the multi-strain probiotic inhibits growth of *L*. *monocytogenes in vivo*. Bioluminescent readings in the ileum after 3.5 h were significantly different for treatment groups (p = 0.0001). Readings recorded for the probiotic treatment group were significantly lower compared to the antibiotic (p = 0.0002) and control (p = 0.0201) groups, but the control and antibiotic treatment groups were similar. The cell numbers of *L*. *monocytogenes* per gram intestine for the duodenum, jejunum, ileum, cecum and colon, 2 and 3.5 h after administration of *L*. *monocytogenes*, is shown in Supplementary Fig. S1. The ileum harboured the highest number of *L*. *monocytogenes* (5–7 log cfu/gram ileum; Supplementary Fig. S1). No significant differences for the log cfu/g intestine were observed for the different GIT sections from the different treatment groups (Supplementary Fig. S1). The multi-strain probiotic inhibited colonization and growth of *L*. *monocytogenes in vivo*, as determined by the Caliper *in vivo* imaging system (IVIS®). However, no cell death of *L*. *monocytogenes* was recorded in the GIT, as determined by standard culturing and plating onto BHI agar (Biolab, Biolab Diagnostics, Midrand, SA) supplemented with 7.5 µg/ml chloramphenicol. Growth inhibition could be due to the production of organic acids, diacetyl, acetoin, hydrogen peroxide and bacteriocins, or through competitive exclusion from the GIT^60,61^.

### Cecum microbiome

**Figure 3:**
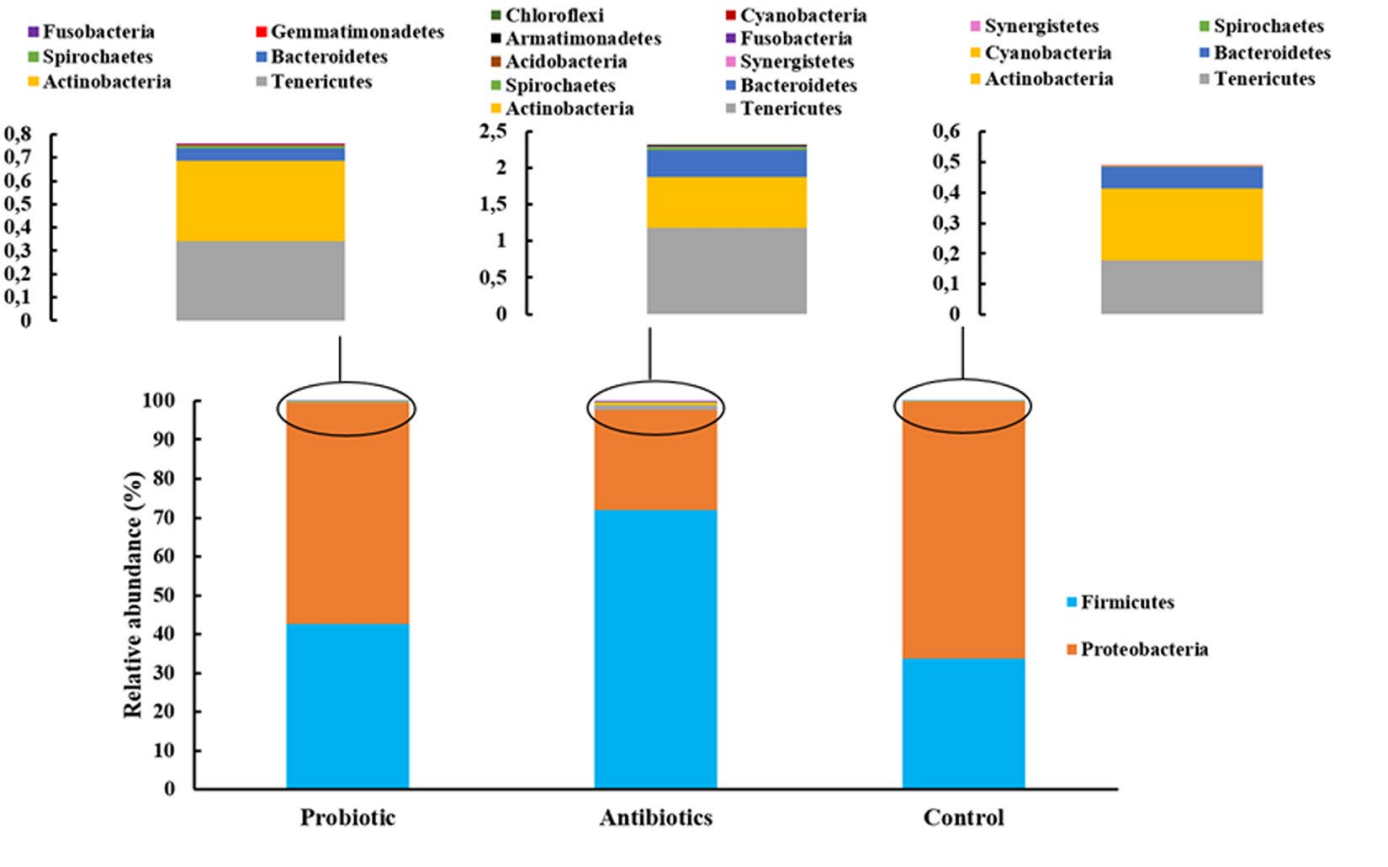
Phyla present in the cecum microbiome of broilers from the different treatment groups i.e. multi-strain probiotic, antibiotic combination and untreated.

**Figure 4:**
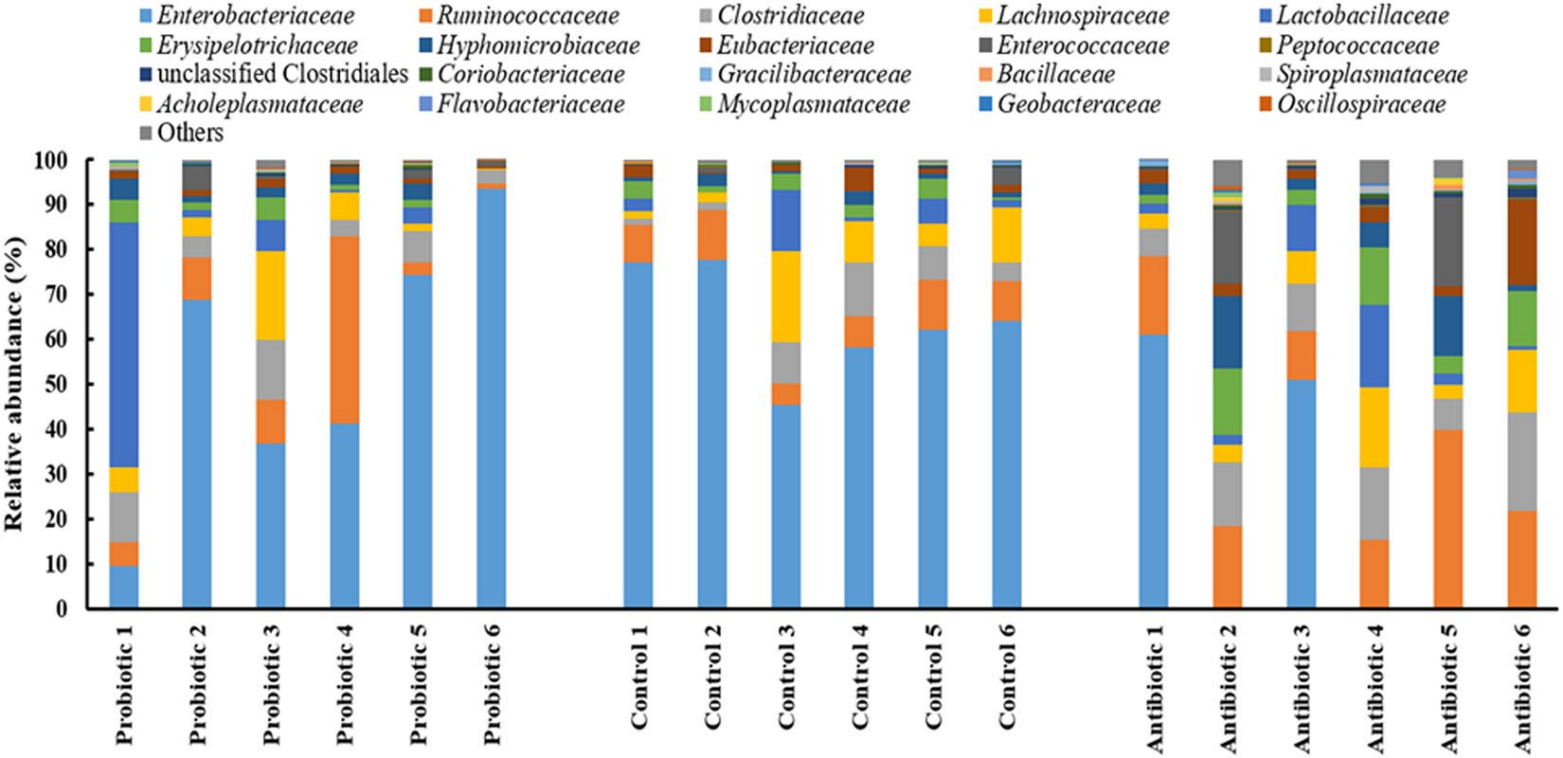
Abundant bacterial families present in the cecum microbiota of broilers from the different treatment groups.

The cecum microbiome grouped into 13 operational taxonomic units (OTU’s), representing the phyla Actinobacteria, Armatimonadetes, Acidobacteria, Bacteroidetes, Chloroflexi, Cyanobacteria, Firmicutes, Fusobacteria, Geminatimonadetes, Proteobacteria, Synergistetes, Spirochaetes, and Tenericutes (Fig. 3). Only two phyla were present at a mean relative abundance of ≥1% and belonged to Proteobacteria (33–72%) and Firmicutes (26–66%). The majority of the Proteobacteria sequences corresponded to sequences recorded for *Enterobacteriaceae* (19–64%) and *Hyphomicrobiaceae* (2–7%). The majority of the Firmicutes sequences correlated with sequences of *Enterococcaceae* (2–6%), *Lactobacillaceae* (4–11%), *Clostridiaceae* (6–13%), *Eubacteriaceae* (1–5%), *Lachnospiraceae* (6–9%), *Ruminococcaceae* (9–21%) and *Erysipelotrichaceae* (2–8%), as shown in Fig. 4.

**Figure 5:**
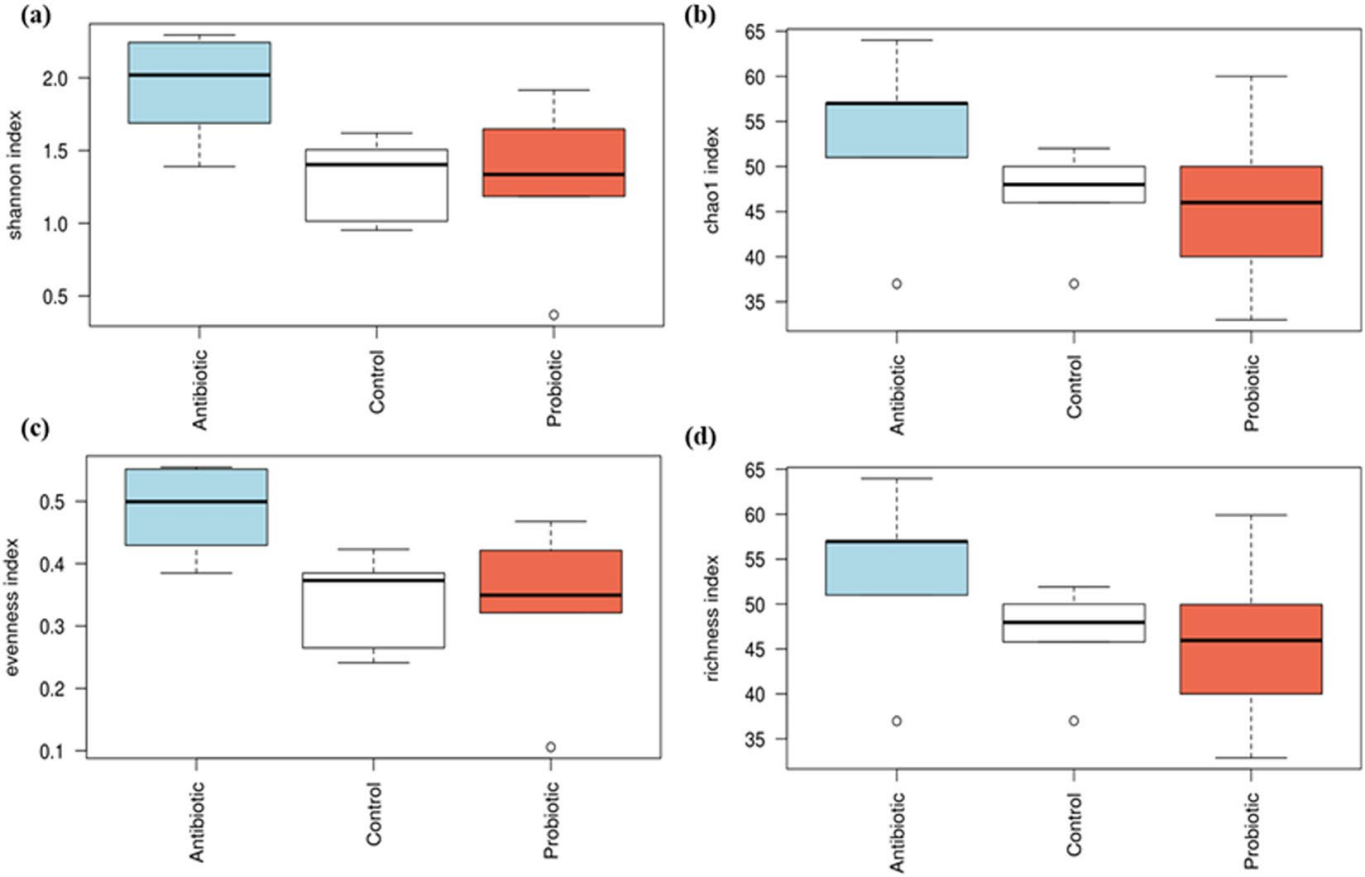
Total species richness obtained by the (**a**) Chao 1 index (ANOVA significance of p = 0.22), the (**b**) Shannon’s diversity index (ANOVA significance of p = 0.02) and the (**c**) richness index (ANOVA significance of p = 0.216) for cecal bacterial communities of broilers from the different treatment group (i.e. multi-strain probiotic, antibiotic combination and untreated).

**Figure 6:**
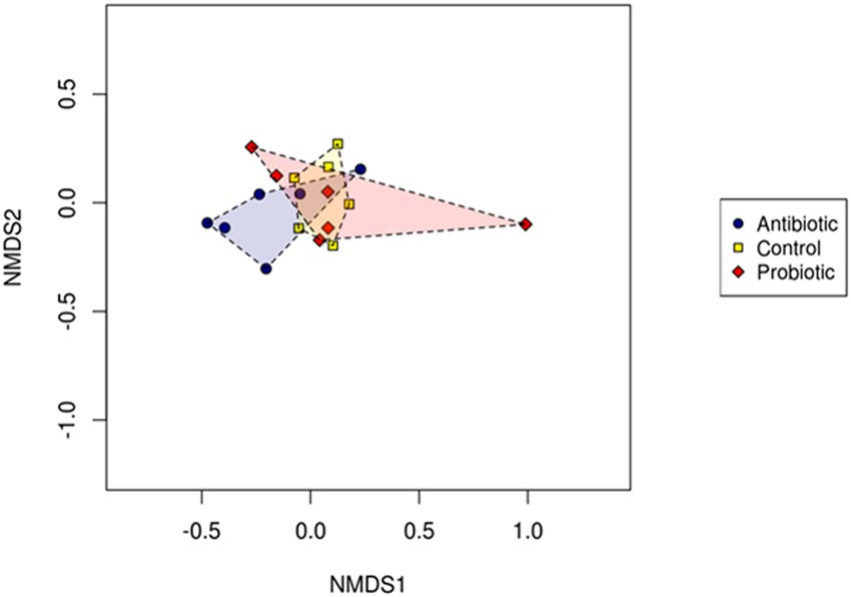
Non-metric multidimensional scaling (NMDS) ordination plot of bacterial communities of the different treatment groups (i.e. multi-strain probiotic, antibiotic combination and control) based on the Bray-Curtis distance.

Chao1 and richness indexes for broilers from the different treatment groups did not differ significantly (Fig. 5). However, the Shannon diversity index (p = 0.019) and evenness index (p = 0.021) differed significantly between the treatment groups. Microbiomes from the antibiotic treatment group were more diverse and OTU’s were, compared to the control and probiotic treatment groups, more evenly distributed. Analysis by mcpHill^62^ revealed that the microbiomes of the control and probiotic treatment groups were similar with respect to rare, average and high abundant species diversity (q = −1, 1, 3; p > 0.05), as shown in Table 4. The antibiotic and control treatment groups differed significantly with respect to average and high abundant species diversity (q = 1, 3; p = 0.028 and p = 0.041 respectively), but did not differ in rare species diversity (q = −1; p > 0.05). The antibiotic and probiotic treatment groups did not differ significantly with respect to rare and high abundant species diversity (q = −1, 3; p > 0.05), but differed significantly with respect to average abundant species diversity (q = 1; p = 0.041). The NMDS plot revealed that microbiomes from the antibiotic treated group formed a cluster separate from the control and probiotic treatment groups (Fig. 6). Adonis analysis revealed significant differences between community composition and treatment (p = 0.029).

**Table 4:** mcpHill diversity analysis of group differences in biodiversity.

**Comparison**	**q (Hill number)**	**p-value**
Control - Antibiotic	−1	0.969
Control - Antibiotic	0	0.639
Control - Antibiotic	1	**0.028** ^*^
Control - Antibiotic	2	**0.035** ^*^
Control - Antibiotic	3	**0.041** ^*^
Probiotic - Antibiotic	−1	0.961
Probiotic - Antibiotic	0	0.521
Probiotic - Antibiotic	1	**0.041** ^*^
Probiotic - Antibiotic	2	0.055
Probiotic - Antibiotic	3	0.065
Probiotic - Control	−1	1
Probiotic - Control	0	1
Probiotic - Control	1	1
Probiotic - Control	2	1
Probiotic - Control	3	1

^*^p < 0.05.

**Figure 7:**
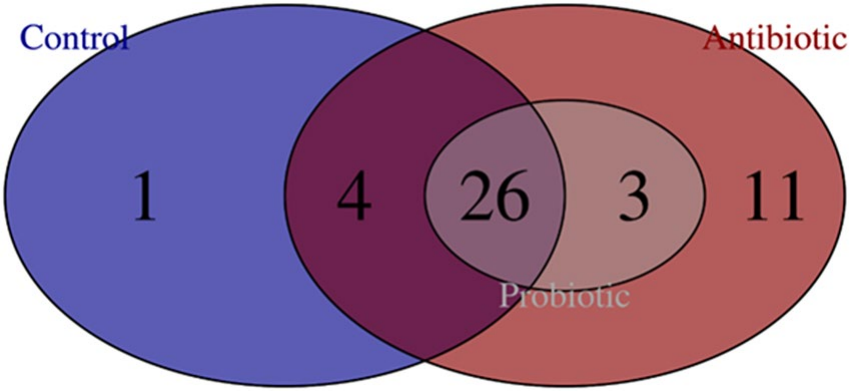
Venn diagram of the core shared bacterial families and unique families present in cecal microbiome of broilers from the different treatment groups (i.e. multi-strain probiotic, antibiotic combination and untreated).

Families present in more than 50% of broilers from a specific treatment were considered part of the microbiome. Microbiomes of broilers from the different treatment groups shared 26 families, i.e. *Geobacteraceae*, *Acholeplasmataceae*, unclassified Clostridiales, *Bacillaceae*, *Clostridiaceae*, Clostridiales Family XI, XIII and XIX Incertae Sedis, *Spiroplasmataceae*, *Ruminococcaceae*, *Planococcaceae*, *Peptostreptococcaceae*, *Peptococcaceae*, *Paenibacillaceae*, *Oscillospiraceae*, *Coriobacteriaceae*, *Mycoplasmataceae*, *Enterobacteriaceae*, *Lactobacillaceae*, *Lachnospiraceae*, *Hyphomicrobiaceae*, *Gracilibacteraceae*, *Veillonellaceae*, *Enterococcaceae*, *Eubacteriaceae* and *Erysipelotrichaceae* (Fig. 7).

Microbiomes from the antibiotic and control treatment groups had four families in common, i.e. *Streptococcaceae* (0.03–0.05%), *Aerococcaceae* (0.007–0.01%), *Anaeroplasmataceae* (0.008–0.03%) and *Xanthomonadaceae* (0.005–0.008%). Genera of *Streptococcaceae* are found in environmental habitats and mammalian hosts, and consists of genera *Streptococcus*, *Lactococcus*, and *Lactovum* ^63^. The family *Aerococcaceae* consists of the genera *Aerococcus*, *Abiotrophia*, *Dolosicoccus*, *Eremococcus*, *Facklamia*, *Globicatella*, and *Ignavigranum* ^64^. Members of this family are present in environmental and clinical habitats^64^. The family *Anaeroplasmataceae* comprises of anaerobic mycoplasmas *Anaeroplasma* and *Asteroleplasma*, commensals of the rumen, with no reported pathogenicity^65^. The family *Xanthomonadaceae* consists of the genera *Xanthomonas*, *Frateuria*, *Fulvimonas*, *Luteimonas*, *Lysobacter*, *Nevskia*, *Pseudoxanthomonas*, *Rhodanobacter*, *Schineria*, *Stenotrophomonas*, *Thermomonas*, and *Xylella* ^66^. Members are typically characterized as environmental microorganisms, with the exception of *Stenotrophomonas* which is infrequently implicated in infections^66^.

The microbiomes of the antibiotic and probiotic treatment groups shared three families, i.e. *Entomoplasmataceae* (0.05–0.26%), *Syntrophomonadaceae* (0.02–0.2%) and *Oceanospirillaceae* (0.005–0.01%). The family *Entomoplasmataceae* comprises the genera *Entomoplasma* and *Mesoplasma* ^67^. Members of *Entomoplasmataceae* have no pathogenicity to their insect or plant host^67^. The family *Syntrophomonadaceae* includes the genera *Candidatus Contubernalis*, *Carboxydocella*, *Dethiobacter*, *Pelospora*, *Syntrophomonas*, *Syntrophothermus*, *Thermohydrogenium* and *Thermosyntropha* ^68^. Members are present in anaerobic environments where organic matter is degraded to methane and carbon dioxide^68^. The family *Oceanospirillaceae* consists of 17 genera, all halotolerant/halophilic, with the exception of *Balneatrix* which has been isolated from freshwater and clinical samples^69^.

The antibiotic treatment group had 11 unique families, i.e. *Pseudomonadaceae* (0.008%), *Staphylococcaceae* (0.008%), *Flavobacteriaceae* (0.3%), Clostridiales Family XIV. Incertae Sedis (0.008%), *Brachyspiraceae* (0.04%), *Demequinaceae* (0.007%), *Desulfuromonadaceae* (0.01%), *Alicyclobacillaceae* (0.06%), *Microbacteriaceae* (0.01%), *Synergistaceae* (0.015%) and *Brucellaceae* (0.008%). The family *Pseudomonadaceae* consists of the genera *Azomonas*, *Azomonotrichon*, *Azorhizophilus*, *Azotobacter*, *Cellvibrio*, *Mesophilobacter*, *Pseudomonas*, *Rhizobacter*, *Rugamonas*, and *Serpens* ^70^. Infection by *P*. *aeruginosa* in broilers is associated with respiratory infections, diarrhoea and septicaemia^71^. The family *Staphylococcaceae* consists of genera *Jeotgalicoccus*, *Macrococcus*, *Nosocomiicoccus*, *Salinicoccus*, *Gemella* and *Staphylococcus* ^63^. *Staphylococcus* members are commensal microorganisms, occasionally causing mastitis in cattle. Major infections associated with genus are due to *S*. *aureus* infections in humans^72^. The family *Flavobacteriaceae* contains more than 90 genera present in a wide variety of habits i.e. water, soil, animals and plants^73^. Many members of the family are capable of digesting macromolecules and polysaccharides^73^. The majority of clostridia present in the GIT of broilers belongs to the family Clostridiales Family XIV Incertae Sedis, with positive traits such as production of butyric acid that promotes a healthy intestinal epithelium^74^. *Brachyspiraceae* has been elevated to the order Brachyspiriales ord. nov.^75^. The family consists of the genera *Brachyspira*, *Exilispira* and *Brevinema* ^76^. Broilers harbour pathogenic *B*. *hyodysenteriae*, *B*. *intermedia*, *B*. *pilosicoli* and *B*. *alvinipulli* and non-pathogenic species *B*. *innocence*, *B*. *murdochii*, and *B*. *pulli* ^77,78^. *Brachyspira* colonizes the large intestine and causes intestinal disease and mortality^76^. The precise significance of *Brachyspira* spp. in birds, species involved, and the epidemiology is not fully understood^76^. The family *Demequinaceae* consists of the genus *Demequina*, and is present in soil and marine environments^79^. The family *Desulfuromonadaceae* contains the genera *Desulfuromonas*, *Desulfuromusa*, *Pelobacter*, *Malonomonas*, and *Geoalkalibacter* ^80^. Members are strictly anaerobic and are found in anoxic environments where they play an important role in the degradation of organic matter and syntrophic associations^80^. None of the members are considered pathogenic^80^. The *Alicyclobacillaceae* family consists of the genera *Alicyclobacillus*, *Kyrpidia*, and *Tumebacillus* ^64^. The family *Microbacteriaceae* consists of numerous genera present in a number of different environments, i.e. terrestrial and aquatic ecosystems, associations with plants, fungi, animals and clinical specimens^81,82^. Several species and subspecies of the family include either plant pathogens, or organisms for which plant pathogenicity has been suggested^81^. The majority of OTU’s were classified to family level, however, members of the genera *Microbacterium* and *Leucobacter* were present. Members of the genus *Microbacterium* are widely distributed in various environments and are associated with plants, insects and clinical specimens^81^. However, little is known about the natural habitats of members of the genus *Leucobacter* ^81^.

The microbiomes of the control treatment group contained one unique family, *Chitinophagaceae* (0.01%). The family *Chitinophagaceae* consists of the genera *Balneola*, *Filimonas*, *Flavisolibacter*, *Gracilimonas*, *Lacibacter*, *Niastella*, *Terrimonas*, *Asinibacterium* and *Chitinophaga* ^83^. Members of this family are found in a range of environments, with some species capable of cellulose hydrolysis^83^.

**Figure 8:**
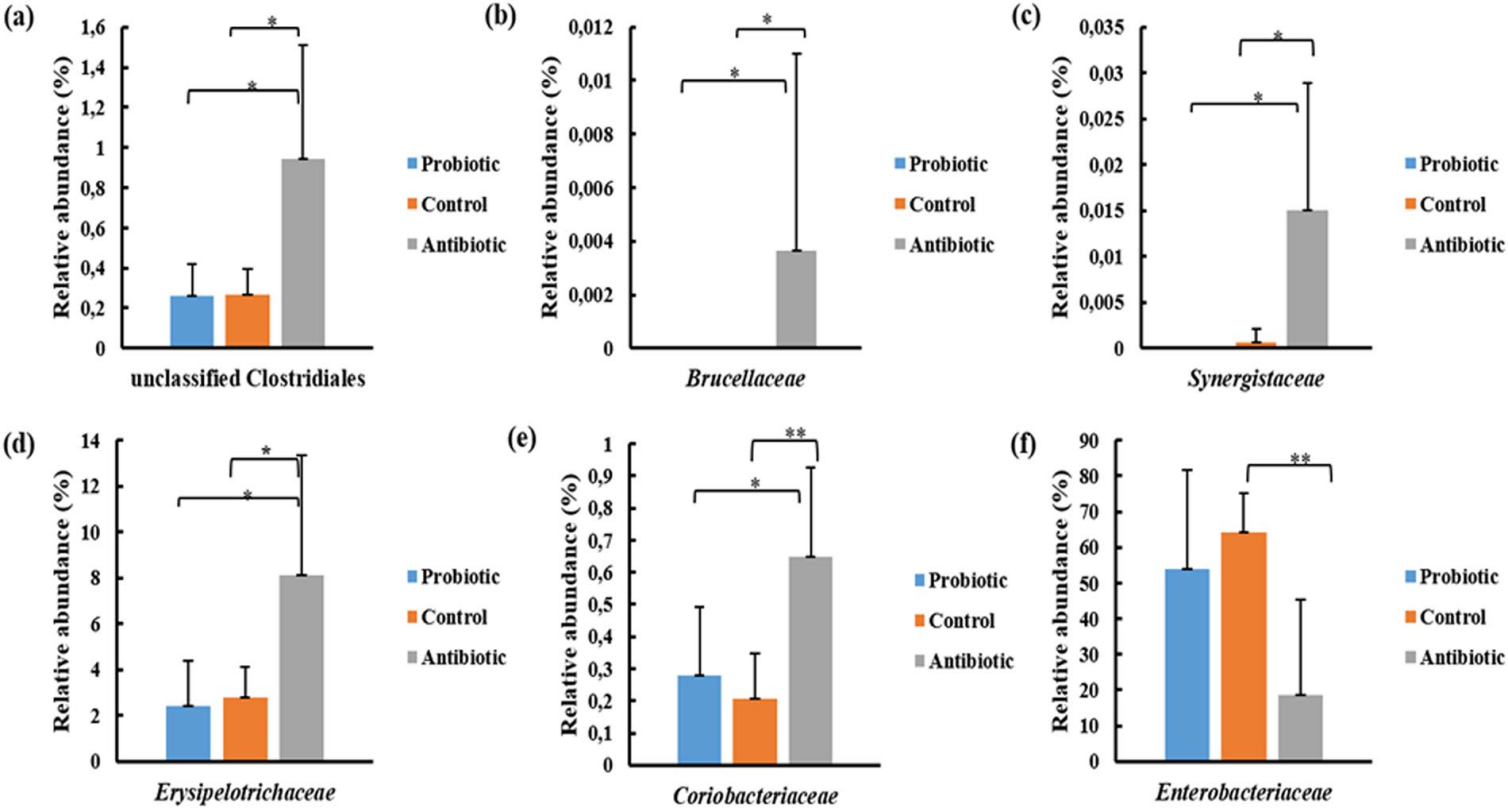
Cecal bacterial families, i.e. (**a**) unclassified Clostridiales, (**b**) *Brucellaceae*, (**c**) *Synergistaceae*, (**d**) *Erysipelotrichaceae*, (**e**) *Coriobacteriaceae* and (**f**) *Enterobacteriaceae* whose abundance significantly differs between the different treatments groups’ i.e. multi-strain probiotic, antibiotic combination and untreated (ANOVA significance, ^*^indicates p < 0.05 and ^**^p < 0.001).

The following families were significantly different (Fig. 8) for the different treatment groups: unclassified Clostridiales (p = 0.011), *Coriobacteriaceae* (p = 0.012), *Synergistaceae* (p = 0.013), *Enterobacteriaceae* (p = 0.018), *Erysipelotrichaceae* (p = 0.026) and *Brucellaceae* (p = 0.033). The antibiotic treatment group had higher levels of unclassified Clostridiales (3.4 fold increase), *Coriobacteriaceae* (2.9 fold increase), *Synergistaceae* (unique family of antibiotic group), *Erysipelotrichaceae* (3.3 fold increase), and *Brucellaceae* (unique family of antibiotic group) and were significantly different from the probiotic (p < 0.05) and control groups (p < 0.05). The families from probiotic and control treatment groups did not differ significantly. The antibiotic group had lower levels of *Enterobacteriaceae* (3.5 fold decrease) and were significantly different (p < 0.05) than the control group. No significant differences were recorded between the antibiotic and probiotic treatment groups, and between the control and probiotic treatment group. Reduction in the levels of *Enterobacteriaceae* is due to the presence of sulphadiazine, trimethoprim and colistin. Sulphadiazine is bacteriostatic with a wide spectrum against Gram-positive and Gram-negative bacteria^84^. Trimethoprim is active against aerobic Gram-positive bacteria (*Staphylococcus*) and aerobic Gram-negative bacteria (*Enterobacter*, *Escherichia*, *Klebsiella* and *Proteus*)^85^. Colistin has bactericidal activity against most Gram-negative aerobic bacilli, i.e. *Acinetobacter*, *Pseudomonas*, *Klebsiella*, *Enterobacter*, *Escherichia*, *Salmonella*, *Shigella* and *Citrobacter* spp.^86^.

The family *Erysipelotrichaceae* comprises the genera *Allobaculum*, *Bulleidia*, *Catenibacterium*, *Coprobacillus*, *Eggerthia*, *Erysipelothrix*, *Holdemania*, *Kandleria*, *Solobacterium* and *Turicibacter* ^87^. Members are highly immunogenic and flourish during post-treatment with broad-spectrum antibiotics^88,89^. *Erysipelotrichaceae* has been correlated to inflammation^89^. Evidence associating members of this family to disease is correlative, and studies examining the impact abundance has on the host is required^90^. The family *Coriobacteriaceae* consists of genera *Adlercreutzia*, *Asaccharobacter*, *Atopobium*, *Collinsella*, *Coriobacterium*, *Cryptobacterium*, *Denitrobacterium*, *Eggerthella*, *Enterorhabdus*, *Gordonibacter*, *Olsenella*, *Paraeggerthella*, *Parvibacter*, and *Slackia* ^91^. They are normal habitants of the mammalian GIT. Members can modulate host metabolism by increased cholesterol absorption^92^, energy metabolism via glycogenesis and enhanced triglycerides synthesis as well as hepatic detoxification pathways^93^, and activation of the isoflavone daidzein a dietary phytoestrogens abundant in soybean^94^. However, several members of *Atopobium*, *Eggerthella*, *Gordonibacter*, *Olsenella*, and *Paraeggerthella* have been implicated in the development of infections, abscesses, intestinal diseases, tumours, periodontitis, vaginosis, and bacteraemia^95^. The majority of OTU’s were identified to family level. However, the genera *Eggerthella*, *Enterorhabdus* and *Gordonibacter* were identified. A decrease in *Coriobacteriaceae* numbers has been correlated to reduced plasma interleukin-6 concentrations and chronic inflammation^96^. The lymphocyte and basophil concentrations for broilers from the antibiotic treatment group were higher at day 19. This could be due to the increase in abundance of *Coriobacteriaceae*. However, knowledge on how and when members of *Coriobacteriaceae* start to become detrimental to the hosts is unknown^91^.

The family *Brucellaceae* (0.008%) was only found in broilers from the antibiotic treatment group. The family consists of the genera *Brucella*, *Crabtreella*, *Daeguia*, *Mycoplana*, *Ochrobactrum*, *Paenochrobactrum*, and *Pseudochrobactrum* ^97^. The majority OTU’s were identified to family level. However, species from the genus *Ochrobactrum* were present. Several *Ochrobactrum* spp. are opportunistic microorganisms and cycle from soil-rhizoplane to immunocompromised individuals^97^. The family *Enterobacteriaceae* consists of 51 genera which includes commensal and pathogenic microorganisms^98^. The majority of sequences could only be classified to family level. However, low levels (0.01–0.4%) of the following genera were present: *Citrobacter*, *Cronobacter*, *Enterobacter*, *Escherichia*, *Shigella*, *Klebsiella*, *Mangrovibacter*, *Pluralibacter*, *Raoutella*, *Salmonella*, *Edwardi*, *Hafnia*, *Trabulsiela* and *Serratia*. *Escherichia*, *Klebsiella*, *Enterobacter*, *Serratia*, *Citrobacter* and *Proteus* are opportunistic pathogens and have been associated with diarrhoea, urinary tract infections, mastitis, arthritis and meningitis^99,100^. Members are generally considered enteric pathogens of animals and some species are associated with a range of diseases^98^. The majority of sequences from the unclassified Clostridiales group were identified to family level. However, the genera *Flavonifractor* and *Pseudoflavonifactor* were identified. The broiler cecum and its mucosal tissue are dominated by Clostridiales^101,102^. Members are known for their conversions of complex polysaccharides to short chain fatty acids such as butyrate which has significant positive effects on growth^103^. However, members are more prominent in inflamed colons, indicating that they may accumulate during the development of colitis^104^. On the contrary, most evidence suggests that the majority of Clostridiales are non-pathogenic, and are beneficial to the host^105^.

The family *Synergistaceae* (0.015%) was only found in broilers from the antibiotic group. Low levels of this family have been reported in the cecum of broilers^106^. *Synergistaceae* inhabit anaerobic environments, i.e. animal gastrointestinal tracts, soil, oil wells, and wastewater treatment plants. In addition, members are present in sites of diseases i.e. cysts, abscesses, gastrointestinal infections and soft tissue infections and are considered opportunistic pathogens^107^. *Fusobacteriaceae*, *Flavobacteriaceae*, *Rhizobiaceae*, *Vibrionaceae*, *Xanthomonadaceae*, *Comamonadaceae*, *Campylobacteraceae* and Clostridiales Incertae Sedis XIII are associated with high feed conversion ratios^108^. *Victivallaceae*, *Synergistaceae*, *Prevotellaceae*, *Rikenellaceae*, *Enterobacteriaceae* and *Ruminococcaceae* are associated with low feed conversion ratios^108^.

A better understanding of the bacterial composition and activity, and the underlying mechanisms by which they modulate the GIT environment, is required to improve the understanding of the role specific bacteria have on the host health and feed utilization^106^. Several studies have investigated the influence that dietary changes has on microbial community structure^109,110^. However, understanding how these changes in bacterial composition relate to metabolic changes, which ultimately relate to improved health and performance needs to be elucidated^106^.

## Conclusions

Supplementation of broiler feed with the antibiotic combination (sulphadiazine, colistin and trimethoprim) or multi-strain probiotic (*L*. *crispatus* DPN167, *L*. *salivarius* DPN181, *L*. *gallinarum* DPN164, *L*. *johnsonii* DPN184, *E*. *faecalis* DPN94 and *B*. *amyloliquefaciens* DPN123) had no effect on the weight gain, feed intake, feed conversion ratio’s, relative lymphoid organ weights, relative gizzard weights, tibia bone parameters and haematological parameters. Broilers from the antibiotic treatment group had higher levels of lymphocytes and basophils counts, and the control group had larger villi area, but these effects were transient and only statistically significant at day 19. Reduced *L*. *monocytogenes* bioluminescence was observed in the ileum of broilers receiving the multi-strain probiotic at 3.5 h after administration of the pathogen. The microbiome of broilers from the antibiotic treatment group had significant lower levels of *Enterobacteriaceae*, and higher levels of unclassified Clostridiales, *Brucellaceae*, *Synergistaceae*, *Erysipelotrichaceae* and *Coriobacteriaceae* in their cecum at day 29. Understanding how these microbiota changes relate to metabolic changes in the host, and the role they play in GIT health and disease needs to be elucidated. While there has been a number of similar studies, information on feed additives is scarce. This study provides basic knowledge required to investigate potential alternatives to antibiotics.

## Materials and Methods

### Birds and housing

The study was approved by the Research Ethics Committee: Animal Care and Use of Stellenbosch University, Stellenbosch (registration number SU-ACUD15–00016). All experiments were performed in accordance with relevant guidelines and regulations. Three-hundred day old as-hatched Cobb 500 broiler chicks were divided into 30 cages of 2 m^2^ each (10 birds per cage) and housed in a temperature controlled poultry rearing house at Mariendahl experimental farm, Stellenbosch University. Each treatment group consisted of 10 cages (100 broilers). Each cage was equipped with feeders and automatic water dispensers. The humidity, temperature and light were controlled according to the Cobb Broiler Management Standards (Cobb Vantress, Colchester, UK) and the South African Animal Welfare Act.

### Bacterial strains and preparation of the probiotic

The multi-strain probiotic consisted of *L*. *crispatus* DPN167, *L*. *salivarius* DPN181, *L*. *gallinarum* DPN164, *L*. *johnsonii* DPN184, *E*. *faecalis* DPN94 and *B*. *amyloliquefaciens* DPN123. Of all bacteria isolated from healthy free-range broilers, strains from these six species were the most resistant to gastric acids and bile, adhered the best to gut epithelial cells and inhibited the growth of *Listeria monocytogenes* and *Salmonella typhimurium in vitro*. The strains were cultured in molasses medium, which consisted of 5.0% (w/v) molasses, 0.3% (w/v) yeast extract, 0.2% (w/v) peptone, 0.004% (w/v) MnSO_4_, 0.001% (w/v) Na-citrate, 0.4% (w/v) K_2_HPO_4_ and 0.02% (v/v) Tween80. The medium was sterilised at 121 °C for 15 min, cooled to 25 °C, the upper phase removed from the sediment and again autoclaved. Thioglycolate (0.15%, w/v) was added to the growth medium of *L*. *crispatus* DPN167 and *L*. *johnsonii* DPN184 to create an anaerobic environment. Incubation was for 3 to 4 days at 37 °C. Cells were harvested (8000 × *g*, 10 min, 4 °C), washed with sterile PBS (0.8%, w/v, NaCl; 0.02%, w/v, KCl; 0.142%, w/v, Na_2_HPO_4_; 0.024%, w/v, KH_2_PO_4_; pH 7.5) and resuspended in sterile cryoprotectant (10%, w/v, lactose and 10.0%, w/v, sucrose, autoclaved at 121 °C for 10 min and cooled to 4 °C). The number of viable cells per gram freeze-dried culture was determined by plating onto MRS Agar (Biolab) or BHI Agar (Biolab). Plates were incubated at 37 °C for 24 h under aerobic and anaerobic conditions. The strains were combined to yield a total cell count of 2.8 × 10^8^ cfu/g freeze-dried powder, consisting of 2.6 × 10^7^ cfu *L*. *crispatus* DPN167, 3.6 × 10^7^ cfu *L*. *salivarius* DPN181, 1.3 × 10^8^ cfu *L*. *gallinarum* DPN164, 1.9 × 10^7^ cfu *L*. *johnsonii* DPN184, 5.1 × 10^7^ cfu *E*. *faecalis* DPN94 and 1.9 × 10^7^ cfu *B*. *amyloliquefaciens* DPN123.

### Feeding trials

The feed contained maize, soya oilcake, sunflower oilcake, canola oilcake, wheat, bran, Ca- phosphate, limestone, salt, lysine, methionine and threonine. The pre-starter was supplied at 178 g per bird (over 7 days). The starter diet was supplied at 354 g per bird (over 7 days), grower diet at 1596 g per bird (over 7 days) and a finisher diet at 1883 g per bird (over 11 days). Feed of broilers from the probiotic treatment group were supplemented with the multi-strain probiotic as follows: pre-starter was supplemented with 24 mg dried probiotic cells per gram feed to yield 6.7 × 10^6^ cfu/gram feed, consisting of 6.1 × 10^5^ cfu *L*. *crispatus* DPN167, 8.4 × 10^5^ cfu *L*. *salivarius* DPN181, 3.1 × 10^6^ cfu *L*. *gallinarum* DPN164, 4.4 × 10^5^ cfu *L*. *johnsonii* DPN184, 1.2 × 10^6^ cfu *E*. *faecalis* DPN94 and 4.4 × 10^5^ cfu *B*. *amyloliquefaciens* DPN123. The starter feed was supplemented with 12 mg probiotic powder per gram feed (3.3 × 10^6^ cfu/gram feed, consisting of 3.1 × 10^5^ cfu *L*. *crispatus* DPN167, 4.2 × 10^5^ cfu *L*. *salivarius* DPN181, 1.6 × 10^6^ cfu *L*. *gallinarum* DPN164, 2.2 × 10^5^ cfu *L*. *johnsonii* DPN184, 6.1 × 10^6^ cfu *E*. *faecalis* DPN94 and 2.2 × 10^5^ cfu *B*. *amyloliquefaciens* DPN123). Grower was supplemented with 5.4 mg probiotic powder per gram feed (1.5 × 10^6^ cfu/gram feed, consisting of 1.4 × 10^5^ cfu *L*. *crispatus* DPN167, 1.9 × 10^5^ cfu *L*. *salivarius* DPN181, 7.0 × 10^5^ cfu *L*. *gallinarum* DPN164, 1.0 × 10^5^ cfu *L*. *johnsonii* DPN184, 2.8 × 10^5^ cfu *E*. *faecalis* DPN94 and 1.0 × 10^5^ cfu *B*. *amyloliquefaciens* DPN123). The finisher was supplemented with 3.5 mg probiotic powder per gram feed (9.9 × 10^5^ cfu/g feed, consisting of 9.0 × 10^4^ cfu *L*. *crispatus* DPN167, 1.3 × 10^5^ cfu *L*. *salivarius* DPN181, 4.4 × 10^5^ cfu *L*. *gallinarum* DPN164, 6.2 × 10^4^ cfu *L*. *johnsonii* DPN184, 1.8 × 10^5^ cfu *E*. *faecalis* DPN94 and 6.5 × 10^4^ cfu *B*. *amyloliquefaciens* DPN123). Average daily intake of the multi-strain probiotic from day 1 to 29 during the different feeding stages is listed in Supplementary Table S6. Broilers from the probiotic treatment group received between 1.0 and 4.1 × 10^8^ cfu daily of the multi-strain probiotic consisting of *Lactobacillus crispatus* DPN167 (9.3 × 10^6^ to 3.8 × 10^7^ cfu), *Lactobacillus salivarius* DPN181 (1.3 × 10^7^ to 5.3 × 10^7^ cfu), *Lactobacillus gallinarum* DPN164 (4.6 × 10^7^ to 1.9 × 10^8^ cfu), *Lactobacillus johnsonii* DPN184 (6.8 × 10^6^ to 2.8 × 10^7^ cfu), *Enterococcus faecalis* DPN94 (1.8 × 10^7^ to 7.5 × 10^7^ cfu) and *Bacillus amyloliquefaciens* DPN123 (6.8 × 10^6^ to 2.8 × 10^7^ cfu).

Broilers in the antibiotic treatment group (10 cages) received the same ration in the four feeding cycles, but the feed was supplemented with a combination of sulphadiazine (0.375 ppm/gram feed), colistin (0.128 ppm/gram feed) and trimethoprim (0.075 ppm/gram feed) and contained no probiotics. Broilers from the antibiotic treatment group received on average between 7.5 to 61.1 ppm sulphadiazine, 2.6 to 20.9 ppm colistin and 1.5 to 12.2 ppm trimethoprim daily for 29 days (Supplementary Table S5). The three antibiotics were selected, as they are often included as feed additives^111^. Broilers in the untreated group (10 cages) served as the control and received feed without antibiotics and probiotics. Lactose and sucrose were added to the feed used in each feeding cycle of the antibiotic and control treatment groups to yield concentrations identical to the feed administered to the probiotic treatment group.

### Health and growth performance

Visual health and growth performance of the birds were evaluated based on daily feed consumption and changes in body mass. Weekly weight and feed intake per pen were recorded and individual weights were calculated as an average of the pen weight. Average feed conversion ratio (FCR) calculated from the feed intake (FI) and body weight gain (BWG). All the birds were weighed and the change in body mass of each cage calculated relevant to the mass recorded on day 1.

### Haematology, organ weight and histology

On days 19 and 29, twenty birds per treatment were randomly selected, euthanized by cervical dislocation and blood collected into K_2_-EDTA tubes by exsanguination. These two days were selected based on the developing stage of the GIT. Previous studies^112^ have shown that at day 19 the GIT is not fully developed, whereas 10 days later, at day 29, the GIT is considered mature. Automated full blood counts were performed using the Celldyne 3700CS haematology analyser (Abbott Diagnostics, Illinois, USA). The number of erythrocytes and their parameters, i.e. haemoglobin content, haematocrit value, mean corpuscular volume, mean corpuscular haemoglobin, mean corpuscular haemoglobin concentration and erythrocyte cell distribution width were determined. The total number of leukocytes was recorded as well as subpopulation counts for heterophils, lymphocytes, monocytes, eosinophils and basophils. Blood platelet (thrombocyte) counts were also recorded.

The spleen and bursa Fabricius of twenty birds per treatment on days 19 and 29, and the gizzards on day 29 were excised and weighed. The gizzards were dissected longitudinally and rinsed under running water before being weighed. The duodenum of 20 broilers per treatment at days 19 and 29 were collected, longitudinally dissected and carefully washed with sterile PBS. The samples were preserved in 10% (v/v) formaldehyde-saline for 30 days, cut to size, placed into embedding cassettes, and processed and impregnated in paraffin wax, using an automated tissue processor TISSUE TEK II 4640B (Miles Laboratories Inc., Naperville, IL). Sections (5 µm in thickness) were prepared with a rotary microtome (Reichert Jung, Heidelberg, Austria), deparaffinised and rehydrated, before staining with haematoxylin and eosin. These sections, prepared as described by Presnell and Schreibman (1997)^113^, were examined using a Nikon SMZ800 (Nikon Corporation, Tokyo, Japan) stereomicroscope, equipped with a 2.5 × magnification objective lens and a Nikon DS-Fi1 digital camera (Nikon Corporation). Images were analysed using ImageJ software (National Institutes of Health, Maryland, USA). Villi height and area were measured from the tip of the villi to the villous-crypt junction for 10 consecutive intact villi. Crypt depth was estimated by measuring 10 crypts per section. Crypt depth was the vertical distance from the villous-crypt junction to the lower limit of the crypt.

### Mineralization of the tibia

The right tibia from the carcasses of twenty birds per treatment at day 29 were cleaned from tissue and cartilage and the dry matter of each determined according to the method described by the Association of Official Analytical Chemists^114^. In short, tibias placed in porcelain cubicles were, dried at 100 °C for 24 h, cooled down for 30 min in a desiccator and then weighed. The tibia were then broken in half, defatted in petroleum for 48 h^115^, dried at 100 °C for 24 h and weighed. Lastly, the tibias were exposed to 600 °C for 24 h and the ash weighed.

### *In vivo* inhibition of *L.monocytogenes*

The ability of the antibiotic and probiotic feed additives in inhibiting colonization and proliferation of *L*. *monocytogenes in vivo* was assessed. At day 14, twelve broilers per treatment group were relocated to the animal housing unit of the Department of Animal Science, Stellenbosch University and each placed in separate cages. Water and feed were supplied *ad libitum*. At day 15, feed was withdrawn 2 h before the administration of *L. monocytogenes* EGDe, a bioluminescent strain obtained from Caliper Life Sciences (Massachusetts, USA). Strain EGDe contains plasmid PL2lux with the *luxABCDE* operon of *Photorhabdus luminescence*. Each of the birds was administered 100 µl (4.28 × 10^8^ cfu) *L. monocytogenes* EGDe by intragastric gavage. Broilers from the probiotic treatment group were administered 100 µl of the multi-strain probiotic (8.34 × 10^8^ cfu) by intragastric gavage, 2 h before the administration of *L*. *monocytogenes* EGDe.

The probiotic preparation was prepared as follows: *L*. *crispatus* DPN167, *L*. *salivarius* DPN181, *L*. *gallinarum* DPN164, *L*. *johnsonii* DPN184 and *E*. *faecalis* DPN94 were cultured in MRS broth for 12 h at 37 °C under anaerobic conditions. *Bacillus amyloliquefaciens* DPN123 was cultured in BHI broth for 12 h at 37 °C under aerobic conditions using an orbital shaker at 100 rpm. Cells were harvested (8000 × *g*, 3 min, 25 °C), washed with two volumes of sterile PBS and resuspended in 100 µl gavage buffer (0.2 M NaHCO_3_ buffer containing 1%, w/v, glucose, pH 8) to yield 8.3 × 10^8^ cfu (5.2 × 10^7^ cfu *L*. *crispatus* DPN167, 6.2 × 10^7^ cfu *L*. *salivarius* DPN181, 1.2 × 10^8^ cfu *L*. *gallinarum* DPN164, 1.3 × 10^8^ cfu *L*. *johnsonii* DPN184, 2.3 × 10^8^ cfu *E*. *faecalis* DPN94 and 2.4 × 10^8^ cfu *B*. *amyloliquefaciens* DPN123). *Listeria monocytogenes* EGDe was cultured in BHI broth (supplemented with 7.5 µg/ml chloramphenicol) under aerobic conditions using an orbital shaker at 100 rpm for 6 h at 37 °C. Cells were harvested (8000 × *g*, 3 min, 25 °C), washed with two volumes of sterile PBS and resuspended in gavage buffer to yield 4.2 × 10^8^ cfu per 100 µl.

After 2 h, and again 3.5 h, of administering *L*. *monocytogenes* EGDe, six broilers per treatment group were euthanized by cervical dislocation. The gastrointestinal tract (GIT) of each bird was dissected longitudinally and screened for the emission of bioluminescence from cells of *L*. *monocytogenes* EGDe by using the Caliper *in vivo* imaging system (IVIS® 100, Caliper Life Sciences). The IVIS was equipped with a cooled charge-coupled-device camera mounted on a light-tight specimen chamber. Exposure was 3 min. Photons emitted at 620 nm were calculated using the software version 3 of Caliper Life Sciences. The values obtained were expressed as photons per second per cm^2^ per steradian (p. S^−1^. cm^−1^. sr^−1^). Regions of interest (ROI) were selected manually. Background bioluminescence was corrected for by overlaying images from intestines with non-bioluminescent bacteria. The GIT of each bird was then dissected to separate the duodenum, jejunum, ileum, ceca and colon. Each section was weighed, homogenized in sterile PBS, serially diluted and plated on BHI agar supplemented with 7.5 µg/ml chloramphenicol. Plates were incubated at 37 °C for 24 h and the number of viable cells expressed as cfu/gram gut.

### Cecal microbiota composition

At day 29 cecal digesta content was collected from six broilers per treatment group and stored at −20 °C. Metagenomic DNA was isolated using the iPrep ChargeSwitch gDNA kit (ThermoFisher, Massachusetts, USA), with a few modifications. One millilitre Tris-HCl buffer (pH 8.0) was added to 200 mg cecal digesta and incubated overnight at 37 °C, in the presence of 50 µl lysozyme (100 mg/ml). Cells were collected (10 000 × *g*, 10 min, 4 °C), suspended in 1 ml ChargeSwitch Lysis Buffer and incubated overnight at 56 °C in the presence of 20 µl of proteinase K (20 mg/ml). DNA was then purified using the iPrep gDNA isolation protocol for AB Library Builder (ThermoFisher) and concentrations assessed using Nanodrop (ThermoFisher) and Qubit readings (ThermoFisher), as per manufacturers’ instructions.

Sequencing of the hypervariable region of the *16 S rRNA* gene was performed using the Ion Torrent 16 S™ Metagenomics Kit (ThermoFisher). DNA (10 ng) was amplified using 16 S primer sets 1 (V2–4–8) and 2 (V3–6, 7–9), and 15 µl Ion Environmental Master Mix in a final volume of 30 µl. Amplification was carried out for 18 cycles, with a 10 min initial denaturation at 95 °C, followed by denaturation at 95 °C for 30 sec, annealing at 58 °C for 30 sec, and elongation at 72 °C for 20 sec. Equal volumes of PCR products were then pooled and purified. Pooled purified amplicons were used to create sequence libraries via the Ion Plus Fragment Library Kit (ThermoFisher) with sample indexing using the Ion Xpress™ Barcode Adapters 1–96 Kit (ThermoFisher). Template preparation was performed using the Ion OneTouch™ 2 System and the Ion S5 OT2 Kit (ThermoFisher). Sequencing was conducted using the Ion S5™ Sequencing reagents on the Ion S5™ system using the Ion 530™ chip. Primary data analysis was performed with Torrent Suite™ Software v4.0 with automated secondary analysis using Ion Reporter™ Software v4.0 (ThermoFisher) and Calypso software^116^. The sequences were deposited on the NCBI SRA databank under Bioproject ID PRJNA352351. The accession numbers are SAMN05971353 (Control 1), SAMN05971354 (Control 2), SAMN05971355 (Control 3), SAMN05971356 (Control 4), SAMN05971357 (Control 5), SAMN05971358 (Control 6), SAMN05971359 (Antibiotic 1), SAMN05971360 (Antibiotic 2), SAMN05971361 (Antibiotic 3), SAMN05971362 (Antibiotic 4), SAMN05971363 (Antibiotic 5), SAMN05971364 (Antibiotic 6), SAMN05971365 (Probiotic 1), SAMN05971366 (Probiotic 2), SAMN05971367 (Probiotic 3), SAMN05971368 (Probiotic 4), SAMN05971369 (Probiotic 5) and SAMN05971370 (Probiotic 6).

### Statistical analyses

GraphPad Prism 6 (GraphPad Software Inc., California, USA) was used to perform statistical analyses. Data of growth performance, gizzard and lymphoid organ weight, histomorphological and haematological parameters, tibia bone weights, viable cell counts and bioluminescent counts were analysed by one-way ANOVA to determine the significance of the main effects and interactions. The mean variances were compared using the Fisher’s LSD test. Differences were considered significant if p values were less than 0.05.

Multidimensionality of biodiversity, various indices of diversity and community composition were calculated and compared using the Calypso software^116^. Alpha diversity was calculated using the shannon index, chao1 index, evenness index and richness index. Alpha diversities were compared using ANOVA analysis. Species diversity was analysed by mcpHill analysis and significant differences determined by the Turkey test^62^. Data was filtered by removing taxa with less than 0.01% abundance and data was normalized by total sum scaling (TSS). Normalization method was applied for downstream analyses i.e. taxa relative abundance, β-diversity, and group significance. Beta diversity was analysed using Bray-Curtis dissimilarity and visualised by nonmetric multidimensional scaling (NMDS) and significant differences between treatment groups determined by Anosim^117^. ANOVA analysis was performed to compare diversity between treatment groups, and pairwise comparison assessed using the student t-test.

## Electronic supplementary material

Supplementary Tables and Figures
