# Supplementary material for: Safety assessment of antibiotic and probiotic feed additives for Gallus gallus domesticus
Source: Sci Rep. 2017 Oct 16;7:12767. doi: 10.1038/s41598-017-12866-7 (PMC5643334; doi:10.1038/s41598-017-12866-7)
Supplement: Supplementary file 1 — Supplementary Tables and Figures [file 41598_2017_12866_MOESM1_ESM.pdf]

Safety assessment of antibiotic and probiotic feed additives for *Gallus gallus domesticus*

D.P. Neveling[1], L. van Emmenes[2], J.J. Ahire[1], E. Pieterse[2], C. Smith[3], L.M.T. Dicks[1]*

[1]*Department of Microbiology,* [2]*Department of Animal Science and* [3]*Department of Physiological Sciences, University of Stellenbosch, Private Bag X1, Matieland 7602, Stellenbosch, South Africa*

Supplementary Tables and Figures

Supplementary Table S1: Mean (± standard deviation) body weight gain (BWG), feed intake (FI), feed conversion ratio (FCR) of broilers from day of hatch to day 28, receiving no treatment, the antibiotic combination and the multi-strain probiotic.

	Day 0-7			Day 0-14			Day 0-21			Day 0-28		
**Treatment**	**BWG**	**FI**	**FCR**	**BWG**	**FI**	**FCR**	**BWG**	**FI**	**FCR**	**BWG**	**FI**	**FCR**
Control	152 ± 12.0	243 ± 13.2	1.61 ± 0.13	438 ± 23.1	579 ± 8.2	1.33 ± 0.08	978 ± 54.4	1566 ± 42.9	1.60 ± 0.06	1675 ± 97.8	2861 ± 169.5	1.72 ± 0.09
Antibiotic	158 ± 20.4	244 ± 14.1	1.58 ± 0.28	437 ± 31.9	576 ± 4.5	1.33 ± 0.10	994 ± 54.4	1570 ± 64.5	1.58 ± 0.07	1766 ± 95.8	2923 ± 62.6	1.67 ± 0.07
Probiotic	160 ± 9.40	245 ± 17.9	1.54 ± 0.13	428 ± 15.6	576 ± 5.0	1.35 ± 0.05	961 ± 59.1	1526 ± 53.3	1.59 ± 0.07	1712 ± 78.6	2935 ± 84.7	1.70 ± 0.04
**p value**	0.509	0.957	0.749	0.644	0.463	0.806	0.443	0.163	0.792	0.112	0.326	0.141

Supplementary Table S2: Heterophil/Lymphocyte ratio (± standard deviation) and thrombocyte concentrations from broilers slaughtered at 19 and 29 days.

Treatment	HET/LYM ratio		Thrombocyte concentration (cell/µl)	
	19 D	29 D	19 D	29 D
Control	0.385 ± 0.351	0.688 ± 0.647	27.8 × 10^3 ± 18.98	47.22 × 10^3 ± 34.68
Antibiotic	0.296 ± 0.192	0.516 ± 0.394	23.03 × 10^3 ± 12.14	44.37 × 10^3 ± 32.99
Probiotic	0.351 ± 0.072	0.431 ± 0.353	41.22 × 10^3 ± 22.66	33.54 × 10^3 ± 20.74
**p value**	0.737	0.357	0.121	0.350

Supplementary Table S3: Relative lymphoid organ weight (± standard deviation) and ratio obtained from broilers slaughtered at 19 and 29 days.

Treatment	Spleen %		Bursa %		Spleen/Bursa ratio	
	19 D	29 D	19 D	29 D	19 D	29 D
Control	0.068 ± 0.018	0.08 ± 0.01	0.247 ± 0.068	0.18 ± 0.03	0.276 ± 0.053	0.49 ± 0.11
Antibiotic	0.069 ± 0.016	0.09 ± 0.02	0.267 ± 0.063	0.16 ± 0.05	0.260 ± 0.046	0.61 ± 0.31
Probiotic	0.062 ± 0.017	0.09 ± 0.02	0.276 ± 0.064	0.17 ± 0.04	0.268 ± 0.058	0.63 ± 0.24
**p value**	0.694	0.559	0.675	0.246	0.853	0.166

Supplementary Table S4: Mean gizzard weight relative to body weight (± standard deviation) obtained from broilers slaughtered at day 29.

Treatment	Body weight (g)	Gizzard weight (g)	Gizzard: Body weight %
Control	1869 (± 149.60)	31.97 (± 4.48)	1.61 (± 0.21)
Antibiotic	1893 (± 158.31)	29.52 (± 2.87)	1.66 (± 0.21)
Probiotic	1873 (± 158.52)	29.39 (± 3.75)	1.58 (± 0.17)
**p-value**	0.882	0.067	0.424

Supplementary Table S5: Mean (± standard deviation) fat free dry bone, ash and the percentage bone ash of tibia obtained from broilers slaughtered at 29 days.

**Treatment**	**Fat free dry bone weight (g)**	**Fat free bone ash weight (g)**	**Fat free bone ash percentage (%)**
Control	3.57 ± 0.40	1.79 ± 0.19	50.27 ± 2.73
Antibiotic	3.52 ± 0.40	1.78 ± 0.19	50.62 ± 3.09
Probiotic	3.41 ± 0.37	1.74 ± 0.21	50.86 ± 1.89
**p value**	0.496	0.721	0.799

Supplementary Table S6: Average daily feed, probiotic and antibiotic consumption rate, by broilers from the probiotic and antibiotic treatment groups.

Feed	Probiotic concentration (cfu/g feed)	Age	Daily average feed consumption (g)	Daily average probiotic consumption (cfu)	Daily average antibiotic consumption (ppm)		
					Sulpha diazine	Colistin	Trimethoprim
**Pre-starter**	6.7×10^6	0<br>1<br>2<br>3<br>4<br>5<br>6<br>7	140	9.4×10^8	52.5	17.9	10.5
**Starter**	3.3×10^6	8	30	2.0×10^8	11.3	3.8	2.3
		9	35	2.3×10^8	13.1	4.5	2.6
		10	39	2.6×10^8	14.6	5.0	2.9
		11	44	2.9×10^8	16.5	5.6	3.3
		12	50	3.4×10^8	18.6	6.4	3.8
		13	55	3.7×10^8	20.6	7.0	4.1
		14	61	4.1×10^8	22.9	7.8	4.6
**Grower**	1.5×10^6	15	67	1.0×10^8	25.1	8.6	5.0
		16	73	1.1×10^8	27.4	9.3	5.5
		17	80	1.2×10^8	30.0	10.2	6.0
		18	86	1.3×10^8	2.3	11.0	6.5
		19	93	1.4×10^8	34.9	11.9	7.0
		20	100	1.5×10^8	37.5	12.8	7.5
		21	107	1.6×10^8	40.1	13.7	8.0
**Finisher**	9.9×10^5	22	115	1.1×10^8	43.1	14.7	8.6
		23	122	1.2×10^8	45.8	15.6	9.2
		24	130	1.3×10^8	48.8	16.6	9.8
		25	137	1.4×10^8	51.4	17.5	10.3
		26	144	1.4×10^8	54.0	18.4	10.8
		27	151	1.5×10^8	56.6	19.3	11.3
		28	157	1.6×10^8	58.9	20.1	11.8
		29	163	1.6×10^8	61.1	20.9	12.2

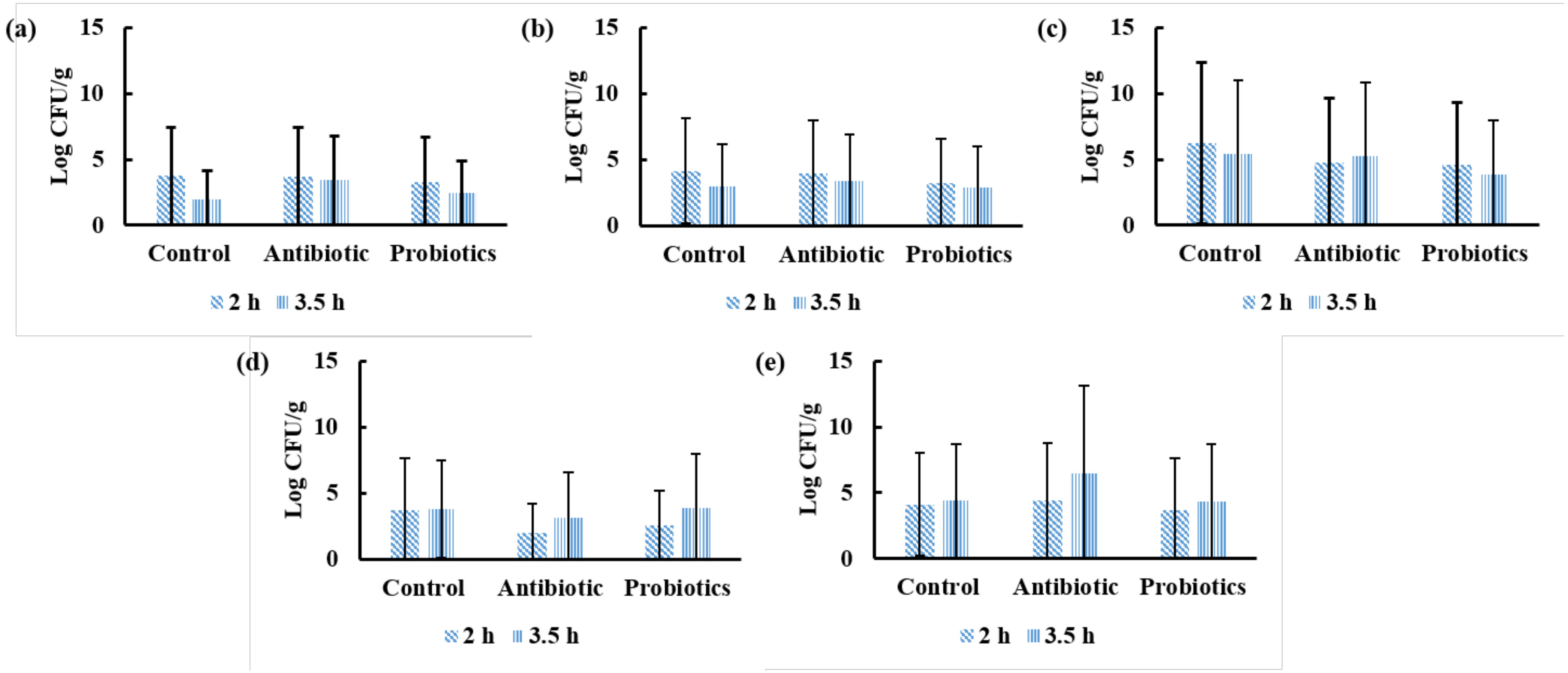

Supplementary Figure S1: Cell numbers (log CFU/ g intestine) of *L. monocytogenes* EGDe recorded in the (a) duodenum, (b) jejunum, (c) ileum, (d) ceca and (e) colon at 2 and 3.5 h after administration of 4.2×10^8 cfu of *L. monocytogenes* EGDe. The $\log_{10}$ averages of the cfu/g intestine were plotted (± standard deviation).
